# 12-Deoxyphorbols Promote Adult Neurogenesis by Inducing Neural Progenitor Cell Proliferation via PKC Activation

**DOI:** 10.1093/ijnp/pyv085

**Published:** 2015-07-29

**Authors:** Noelia Geribaldi-Doldán, Eugenia Flores-Giubi, Maribel Murillo-Carretero, Francisco García-Bernal, Manuel Carrasco, Antonio J. Macías-Sánchez, Jesús Domínguez-Riscart, Cristina Verástegui, Rosario Hernández-Galán, Carmen Castro

**Affiliations:** Area de Fisiología, Facultad de Medicina, Universidad de Cadiz, Cádiz, Spain (Mrs Geribaldi-Doldán, Mrs Flores-Giubi, Dr Murillo-Carretero, Mr García-Bernal, Dr Carrasco, Dr Domínguez-Riscart, and Dr Castro); Departamento de Química Orgánica, Facultad de Ciencias, Universidad de Cádiz, Puerto Real, Cádiz, Spain (Mrs Flores-Giubi, Dr Macías-Sánchez, and Dr Hernández-Galán); Departamento de Anatomía y Embriología Humana, Universidad de Cádiz, Cádiz, Spain (Dr Verástegui).

**Keywords:** 12-deoxyphorbol, prostratin, PKC, neurogenesis, neural progenitor cells

## Abstract

**Background:**

Neuropsychiatric and neurological disorders frequently occur after brain insults associated with neuronal loss. Strategies aimed to facilitate neuronal renewal by promoting neurogenesis constitute a promising therapeutic option to treat neuronal death-associated disorders. In the adult brain, generation of new neurons occurs physiologically throughout the entire life controlled by extracellular molecules coupled to intracellular signaling cascades. Proteins participating in these cascades within neurogenic regions constitute potential pharmacological targets to promote neuronal regeneration of injured areas of the central nervous system.

**Methodology:**

We have performed in vitro and in vivo approaches to determine neural progenitor cell proliferation to understand whether activation of kinases of the protein kinase C family facilitates neurogenesis in the adult brain.

**Results:**

We have demonstrated that protein kinase C activation by phorbol-12-myristate-13-acetate induces neural progenitor cell proliferation in vitro. We also show that the nontumorogenic protein kinase C activator prostratin exerts a proliferative effect on neural progenitor cells in vitro. This effect can be reverted by addition of the protein kinase C inhibitor G06850, demonstrating that the effect of prostratin is mediated by protein kinase C activation. Additionally, we show that prostratin treatment in vivo induces proliferation of neural progenitor cells within the dentate gyrus of the hippocampus and the subventricular zone. Finally, we describe a library of diterpenes with a 12-deoxyphorbol structure similar to that of prostratin that induces a stronger effect than prostratin on neural progenitor cell proliferation both in vitro and in vivo.

**Conclusions:**

This work suggests that protein kinase C activation is a promising strategy to expand the endogenous neural progenitor cell population to promote neurogenesis and highlights the potential of 12-deoxyphorbols as pharmaceutical agents to facilitate neuronal renewal.

## Introduction

Neuropsychiatric and neurodegenerative diseases frequently occur after central nervous system (CNS) insults such as stroke or traumatic brain injury, characterized by an irreversible neuronal loss, for which there is no adequate treatment at present. Among novel treatment approaches, some intend to promote neuronal replacement in the injured tissue through the generation of new neurons from neural stem cells (NSCs). In mammals, adult NSCs continuously undergoing physiological neurogenesis are located fundamentally in the dentate gyrus of the hippocampus (DG) and the subventricular zone (SVZ). Glial-like NSCs within these regions produce multipotent undifferentiated neural progenitor cells (NPCs), which can commit to either a neuronal phenotype (through neuroblast and mature neuron production), or a glial phenotype, generating either astrocytes or oligodendrocytes (Alvarez-Buylla and Garcia-Verdugo, 2002; Goldman, 2003). Cells isolated from these areas, and cultured in the presence of growth factors such as epidermal growth factor (EGF) and/or basic fibroblast growth factor (bFGF or FGF-2), generate cell aggregates known as neurospheres that contain NPCs, which exhibit stem cell properties, including the ability to proliferate, self-renew, and undergo multipotential differentiation, generating neurons, astrocytes, and oligodendrocytes (Pastrana et al., 2009). Newly generated neurons have been found in brain lesions of different origins (Parent et al., 1997; Liu et al., 1998; Jin et al., 2001; Luka et al., 2002), some of them resulting from the differentiation and maturation of neuroblasts generated in neurogenic regions that had migrated to the injured area and some others generated from nearby stem cells activated locally by the specific injury. However, the neuroregenerative capacity under any of these conditions is very limited (Arvidsson et al., 2002; Nakatomi et al., 2002; Teramoto et al., 2003); thus, to improve neuron replacement within an injured region of the CNS, it is necessary to find molecules that activate proliferation and/or neuronal differentiation of NPCs and NSCs.

Growth factors like EGF or bFGF promote proliferation and/or fate determination on NPC pools (Palmer et al., 1995; Doetsch et al., 2002) through interaction with receptor tyrosine kinases (Ullrich and Schlessinger, 1990; Annenkov, 2014). Receptor tyrosine kinases activate intracellular signaling molecules, including the family of protein kinase C (PKC) (Annenkov, 2014); several members of this PKC family are expressed in neurogenic regions (Minami et al., 2000) and participate in various signaling cascades initiated by growth factors, often determining growth factor specificity (Corbit et al., 2000). Ten serine-threonine kinases constitute the PKC family, which are divided into 3 subfamilies: the classical (α, β_1_, β_2_, and γ), the novel (δ, ε, θ, and η), and the atypical (ζ and λ). Atypical PKCs ζ and λ have been involved in NSC to neuron transition (Wang et al., 2012), and several other isoforms of PKC are expressed in NPCs isolated from the SVZ of newborn rats, among which PKCε seems to be relevant for astrocytic differentiation (Steinhart et al., 2007). It is plausible that particular isoforms of PKC may be implicated in other specific aspects of adult neurogenesis, like NPC autoregeneration, proliferation, survival, or neuronal differentiation.

Phorbol esters are natural, plant-derived diterpenes known to activate novel and classical PKCs by binding to their regulatory domain, mimicking their physiological activator diacylglicerol. The most common phorbol ester is phorbol-12-myristate-13-acetate (PMA) (Goel et al., 2007), whose major drawback is its strong tumor-promoting activity (Deleers and Malaisse, 1982). Other naturally occurring phorbols, like prostratin, activate PKC but lack the tumor-promoting activity that makes PMA unsuitable for biomedical research (Szallasi and Blumberg, 1991b); rather than that, prostratin has shown some tumor-supressing activity (Shen et al., 2015), but it is best known for its antiviral properties, particularly against HIV (see Miana et al., 2015 for a recent review). Prostratin is a 12-deoxyphorbol first isolated from the plant *Pimelea prostrate*, an endemic New Zealand shrub. Extracts from this and other plants containing 12-deoxyphorbols have been historically used in Asian medicine. Diterpenes are also abundant in plants of the *Euphorbiaceae* family.

We have studied herein the effect of PKC activation by PMA and prostratin on the proliferation and expansion of NPC in vitro and in vivo. In addition, we have isolated 6 natural products from *Euphorbia resinifera* with a 12-deoxyphorbol structure similar to that of prostratin, and have comparatively tested this library of compounds on NPC proliferation, in an attempt to identify whether additional harmless molecules were capable of promoting neurogenesis and improving neuronal replacement. Our results show that prostratin as well as the related compound ER272 increase NPC proliferation in a PKC-dependent manner, both in vivo and in vitro, without affecting the neuronal differentiation potential of these NPCs.

## Materials and Methods

### Reagents

12-Deoxyphorbols were isolated and purified in our laboratory. PMA (13-*O*-acetyl-12-*O*-tetradecanoylphorbol), prostratin (13-*O*-acetyl-12-deoxyphorbol), and other products unless otherwise indicated were purchased from Sigma-Aldrich (St. Louis, MO). The general PKC inhibitor bisindolylmaleimide I, or G06850, was from Calbiochem (Millipore, Billerica, MA), used at a final concentration of 0.5 µM, and added to the cells 30 minutes before addition of PKC activators. Stock solutions of all PKC interacting compounds were prepared in dimethyl sulfoxide (DMSO) and prediluted in culture medium before addition to cell cultures.

### Plant Material

Latex from *Euphorbia resinifera* was collected in Tanat, Morocco, on December, 2007, by making cuts on stalks and collecting the milky secretion. Natural products with 12-deoxyphorbol structures were isolated from this latex as described in the supplementary Methods.

### Animal Subjects

Seven-day postnatal CD1 mice were used for NPC isolation from the SVZ. Two-month-old adult male mice were used for intracerebroventricular (ICV) injections (in vivo experiments). Animals were housed under controlled conditions of temperature (21–23°C) and light (light:dark 12:12) with free access to food (AO4 standard maintenance diet; SAFE, Épinay-sur-Orge, France) and water. Care and handling of animals were performed according to the Guidelines of the European Union Council (2010/63/EU) and following the Spanish regulations (65/2012 and RD53/2013) for the use of laboratory animals.

### SVZ Cell Isolation and Culture

NPCs were obtained from the SVZ of 7-day postnatal mice following the same procedure described in Rabaneda et al. (2008). Neurosphere cultures were maintained in defined medium (DM) composed of Dulbecco’s modified Eagle’s medium/F12 medium (1:1vol/vol) plus 1mg/L gentamicin (GIBCO) and the B27 supplement (Invitrogen, Carlsbad, CA). EGF (20ng/mL) and bFGF (10ng/mL; both from PeproTech, Frankfurt, Germany) were added to DM for culture expansion, but only bFGF was present during experimental settings, unless otherwise indicated.

### Neurosphere Assay

To test the effects of our compounds on NPC proliferation, single cells from mechanically disaggregated neurospheres were plated onto anti-adherent 96-well plates at a density of 20,000 cells/mL in DM + 10ng/mL bFGF. 12-Deoxyphorbols and other pharmacological agents were added at the time of seeding, and all conditions were run in triplicates.

Neurosphere number and size were measured as previously described (Rabaneda et al., 2008). Results were obtained from a minimum of 3 independent experiments performed with triplicate samples.

### Cell Viability Assay

Cells were seeded at a density of 20,000 cells/mL and cultured in the absence or presence of specific treatments. Then, cultures were disaggregated and mixed with trypan blue [0.04% wt/vol in phosphate-buffered saline (PBS)]. Viable cells (those excluding the trypan blue dye) were counted using a hemocytometer under an inverted microscope and were expressed as percentage of the total number of cells.

### Immunocytochemistry

Neurosphere cells were disaggregated and adhered onto poly-L-ornithine-coated 8-well glass slide chambers (Lab-Tek) and were grown for 48 hours in the presence of bFGF plus specific treatments. Immunodetection was performed as previously described (Rabaneda et al., 2008). Antibodies used were rabbit polyclonal anti-Ki67 (1:1000; Vector) and goat anti-rabbit IgG labeled with Alexa Fluor 594 (1:1500; Invitrogen, Carlsbad, CA). Nuclei were counterstained for 10 minutes with 0.1mg/L DAPI (4',6-diamidino-2-phenylindole). Ki67^+^ cells were counted under a BX60 epifluorescence microscope (Olympus, Hamburg, Germany) and expressed as percentage of the total number of cells, determined by DAPI staining. Quantification was performed in 12 predetermined visual fields/well and 3 wells/condition. Experiments were repeated a minimum of 3 times and results were expressed as the mean ± SEM.

### Cell Death Measurement

Cell death was estimated by counting pyknotic nuclei in the same sample preparations and wells used for immunocytochemistry (see previous paragraph). Nuclei showing chromatin hypercondensation (apoptotic and/or necrotic) were detected by DAPI staining and expressed as percentage of the total number of nuclei.

### Western Blot

Cells from disaggregated neurospheres were resuspended in defined medium without any added growth factor and seeded in 12-well plates (500000 cells per well and condition). Immediately after, cells were pretreated with or without 0.5 µM G06850 for 30 minutes, then with or without 5 µM prostratin and, 1.5 hours later, stimulated with or without 10ng/mL bFGF. Western blot was performed as previously described (Carrasco et al., 2014). Antibodies used were mouse anti-cyclin D1, rabbit anti-cyclin E (both at 1:500, Santa Cruz Biotechnologies, Santa Cruz, CA), and mouse anti-β-actin as loading control (1:1000, Sigma-Aldrich, St Louis, MO). Secondary antibodies were from commercial kits (Western Breeze, Invitrogen, Carlsbad, CA). Band density was quantified using the ImageJ software.

### ICV Administration of Prostratin, ER272, and ER271

Adult male mice weighing 35 to 40g were anaesthetized with ketamine/xylazine and placed on a stereotaxic frame (Kopf Instruments). A small trepanation was made 0.8mm lateral to Bregma, where the needle of a 5-µL Hamilton syringe was introduced 2.4mm below the brain surface. Ultra-filtered prostratin, diluted in PBS at a concentration of 5 µM (n=12), or vehicle (n=12), were injected ipsilaterally into the right lateral ventricles. Single injections of 2 µL per brain were performed through a time-frame of 10 minutes (corresponding to a total of 4ng of prostratin in the case of prostratin-injected mice); afterwards, the Hamilton syringe was left another 5 minutes before removal. Animals received daily injections of bromodeoxyuridine (BrdU) (120mg/kg), during 3 days starting the day of the surgical procedure. Then, mice were deeply anaesthetized with pentobarbital and perfused with 4% parafolmadehyde via ascending aorta. Brains were removed and sliced in 30-µm serial sections from Bregma 2.1 to -3.8 (Franklin and Paxinos, 2007), where the SVZ and hippocampus are contained. In a different set of experiments, the same procedure was followed except that mice were injected with 2 µL of 1 µM ER272 (n=6) or 1 µM ER271 (n=6), corresponding to a dosage of 0.8ng of each compound per brain, and 6 vehicle-injected mice were processed in parallel.

### Brain Processing and Immunohistochemistry

Brain removal, processing, and immunohistochemical techniques to detect BrdU and doublecortin (DCX) were performed as previously described (Rabaneda et al., 2008). Antibodies used were mouse monoclonal anti-BrdU (1:100) from Dako (Hamburg, Germany), goat polyclonal anti-DCX (1:200, Santa Cruz Biotech.), AlexaFluor 488 donkey anti-mouse and AlexaFluor 594 (both at 1:1000, from Life Tech.), and biotinylated anti-mouse IgG (1:250) from GE Healthcare (Albany, NY).

### Quantification of Neurogenesis in Brain Sections

BrdU^+^ and DCX^+^ cells were counted in neurogenic areas as previously described (Rabaneda et al., 2008; Carrasco et al., 2014).

### Statistical Analysis

When more than one treatment group were compared, statistical analyses were performed using 1-way ANOVA, and when normality failed a nonparametric Dunnett’s Method was performed. The Student’s *t* test was used when only one treatment group was compared with the control. Differences were considered significant at values of *P*≤.05.

## Results

### PMA-Induced PKC Activation Promotes NPC Proliferation

NPCs divide in culture in the presence of growth factors, forming floating cell aggregates known as neurospheres (Bull and Bartlett, 2005). The size reached by neurospheres at specific time-points is indicative of NPC proliferation, while the number of new neurospheres formed after subsequent subcultures depends on NPC self-renewal capacity (Ramirez-Castillejo et al., 2006) and survival (Torroglosa et al., 2007). We used neurosphere assays, in which the size and number of newly formed neurospheres is estimated, to test whether PKC activators modulated NPC proliferation. Experiments were performed in the presence of a single growth factor, bFGF, which should not mask other mitotic stimuli.

**Figure 1.**
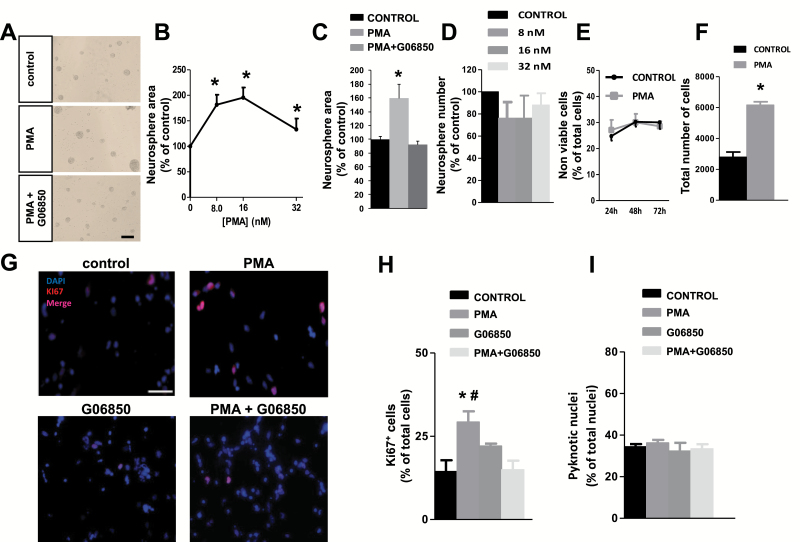
Effect of phorbol-12-myristate-13-acetate (PMA) on neural progenitor cell (NPC) proliferation in vitro. Proliferation was tested in NPC cultures grown in the presence of the growth factor basic fibroblast growth factor (bFGF), unless otherwise indicated. (A) Phase-contrast microscopy images of neurospheres cultured for 72 hours with or without the protein kinase C (PKC) activator PMA (16nM) alone or in combination with the PKC inhibitor G06850 (0.5 µM). Scale bar indicates 200 µm. (B) Graph shows the effect of increasing concentrations of PMA on neurosphere area after 72 hours of culture. (C) Graph shows neurosphere area after 72 hours of treatment with 16nM PMA, alone or in combination with 0.5 µM G06850. (D) Effect of increasing concentrations of PMA on neurosphere number, counted after 72 hours of culture. (E) Effect of 16nM PMA on cell viability. (F) Effect of 16nM PMA on total cell number, counted in a Neubauer chamber after neurosphere disaggregation. (G) Fluorescence microscopy images of neurosphere-derived adhered cells grown for 48h with or without 16nM PMA and/or the PKC inhibitor G06850 (0.5 μM). Cells were immunostained to detect the proliferation marker Ki67 and nuclei were counterstained with 4',6-diamidino-2-phenylindole. Scale bar indicates 100 µm. (H) Quantification of Ki67^+^ cells in cultures described in G. (I) Quantification of pyknotic nuclei in cultures described in G. *P<.05 compared with control in a Student’s *t* test; #*P*<.05 compared with the other groups by 1-way ANOVA.

NPC were treated for 72 hours with increasing concentrations of PMA, a potent and widely known PKC activator. PMA treatment increased neurosphere size, being 16nM, the most effective concentration (Figure 1 A-B), and this effect was reverted by the PKC inhibitor G06850 (Figure 1 C). PMA treatment did not modify neurosphere number (Figure 1D). These results suggested that PMA promoted NPC proliferation without affecting the viability of neurosphere-forming cells (as neurosphere number was unchanged). Neurosphere enlargement in cultures treated with 16nM PMA for 72 hours was accompanied by an increment in the total number of cells (Figure 1F), while cell viability was not affected by this concentration of PMA (Figure 1E). The proliferative effect of PMA was confirmed by immunocytochemical detection of the cell cycle-marker Ki67 in neurosphere-derived cells that had been cultured onto an adherent surface for 48 hours. In the presence of 16nM PMA, a 2-fold increase in the percentage of Ki67^+^ cells was observed compared with the control, and pretreatment of cultures with the PKC inhibitor G06850 (0.5 µM) abolished the effect of PMA (Figure 1 G-H); these results indicate that PMA induced NPC proliferation by activating PKC. The percentage of pyknotic nuclei was not affected by any treatment (Figure 1I).

### Effect of Prostratin-Induced PKC Activation on NPC Proliferation

**Figure 2.**
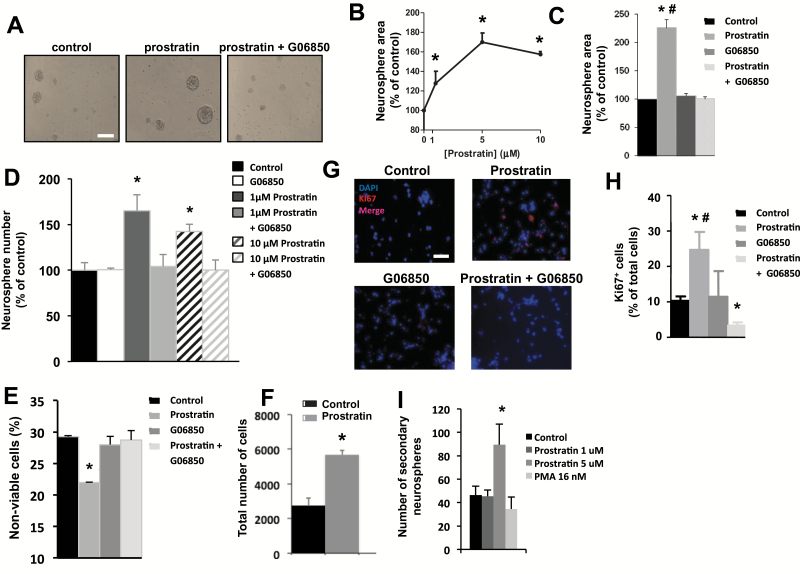
Effect of prostratin on neural progenitor cell (NPC) proliferation in vitro. Proliferation was tested in NPC cultures grown in the presence of basic fibroblast growth factor (bFGF). (A) Phase-contrast microscopy images of neurospheres cultured for 72 hours in the absence or presence of prostratin (5 µM) with or without addition of the PKC inhibitor G06850 (0.5 µM). Scale bar indicates 200 µm. (B) Graph shows the effect of increasing concentrations of prostratin on neurosphere area after 72 hours of culture. (C) Graph shows neurosphere area after 72 hours of treatment with or without 5 µM prostratin and/or 0.5 µM G06850. (D) Effect of prostratin on neurosphere number, counted 72 hours after seeding, in the absence or presence of 0.5 µM G06850. (E) Percentage of nonviable cells quantified by trypan blue exclusion after 48 hours of culture in the presence or absence of 5 µM prostratin and/or 0.5 µM G06850. (F) Total cell number in NPC cultures after 72 hours in the presence or absence of 5 µM prostratin. (G) Fluorescence microscopy images of neurosphere-derived adhered cells grown for 48 hours with or without 5 µM prostratin and/or 0.5 µM G06850. Cells were immunostained to detect Ki67, and nuclei were counterstained with 4’,6-diamidino-2-phenylindole. Scale bar indicates 100 µm. (H) Quantification of Ki67^+^ cells in cultures described in G. (I) Effect of a 72-hour pretreatment of NPC with prostratin or PMA (at the indicated concentrations) on the number of secondary neurospheres formed in the following 72-hour subculture in the absence of drugs. *P<.05 compared with control in a Student’s *t* test; #*P*<.05 compared with the other groups in ANOVA for repeated measurements, followed by Student’s *t* test.

In contrast to PMA, other phorbols like prostratin are nontumorigenic PKC activators (Szallasi and Blumberg, 1991a, 1991b; Szallasi et al., 1992). Thus, the effect of this compound on NPC proliferation was tested. In the presence of 1 to 10 µM prostratin, neurosphere size increased in a concentration-dependent manner, reaching its maximum at 5 µM (Figure 2A-B). The effect of prostratin on neurosphere size occurred through PKC activation, because it was reverted by pretreatment with the PKC inhibitor G06850 (Figure 2A, C). In contrast to PMA, PKC activation mediated by prostratin increased the number of neurospheres formed (Figure 2D); this effect of prostratin could be due to increased NPC survival but could also be indicative of a higher number of neurosphere-forming cells dividing actively. In fact, treatment with prostratin for 72 hours not only increased the total cell number present in neurospheres, but it also had a protective effect on cell viability (Figure 2E-F). Also, treatment of adhered NPC cultures with prostratin induced a 2.5-fold increase in the percentage of Ki67^+^ cells, which was reduced by pretreatment with the PKC inhibitor G06850 even below control levels (Figure 2G-H), confirming that prostratin-induced activation of PKC was responsible for this rise in the number of cycling cells. No effect of prostratin on pyknotic nuclei percentage was observed (data not shown).

A major drawback of manipulating NPC proliferation may be the loss of self-renewal capacity, causing stem-cell pool exhaustion. In the context of NPC cultures, this would result in a decrease on neurosphere number as subcultures go on (Ramirez-Castillejo et al., 2006). To rule out that PKC activation was affecting NPC self-renewal, we disaggregated neurospheres that had been treated for 72 hours with prostratin, PMA, or none, reseeded single cells at a density of 20000 cells/mL in fresh control medium, and cultured them for another 72 hours. The number of newly formed secondary neurospheres was then counted. Surprisingly, pretreatment with 5 µM prostratin increased the number of secondary neurospheres formed in the subsequent subculture when compared with the control, while no effect of PMA was observed (Figure 2I).

The other concern that had to be ruled out was whether the capacity of NPCs to differentiate, particularly towards the neuronal lineage, would be compromised after treatment with PKC activators. To answer this, we disaggregated neurospheres that had been treated for 72 hours with prostratin, PMA, or none and seeded them onto adherent surfaces in differentiating conditions for another 72 hours. Then, the neuronal marker β-III-tubulin and the glial marker GFAP were detected by immunocytochemistry. Prostratin did not change the percentage of cells committed to either neuronal or glial differentiation, while only a small effect of PMA on glial differentiation was observed (supplementary Figure S1; supplementary Methods).

### Prostratin and bFGF Work in Synergy to Upregulate Cyclin Expression in NPC Cultures

Growth factors like EGF or bFGF induce NPC proliferation through activation of Akt and Erk1/2 signaling pathways (Sung et al., 2007; Torroglosa et al., 2007), which results in cyclin upregulation and cell division (Rabaneda et al., 2008). To study whether these mechanisms were involved in the proliferative effect of prostratin, a neurosphere assay was performed in the absence or presence of the Akt pathway inhibitor LY294002 or the Erk1/2 pathway inhibitor UO126. The effect of prostratin on neurosphere growth was blocked by UO126 and, remarkably, completely abolished by LY294002, indicating that both Akt and Erk1/2 are essential downstream effectors of prostratin’s action (supplementary Figure S2).

**Figure 3.**
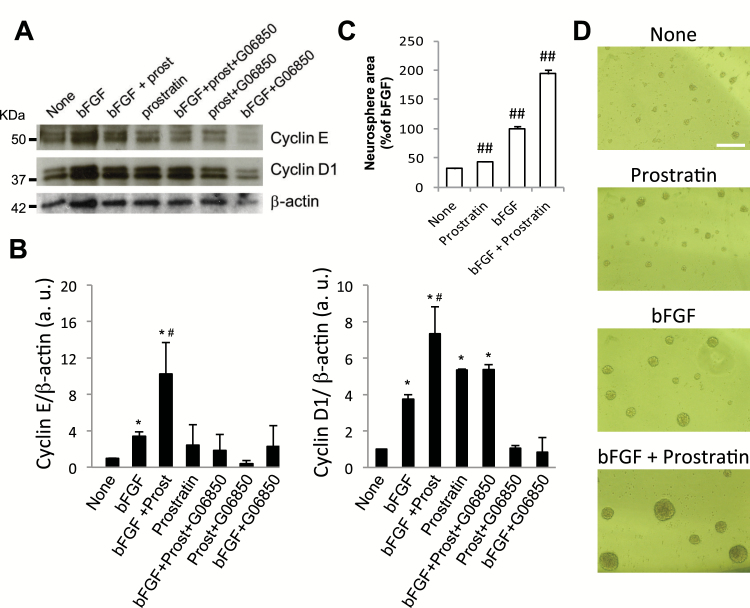
Effect of prostratin on the expression of cyclins. (A) Representative images of Western blots showing immunodetection of cyclin D1 and E, and beta-actin as a loading control. Neural progenitor cell (NPC) cultures were pretreated with or without prostratin (5 μM) and/or the PKC inhibitor G06850 (0.5 μM) for 1.5 hours and then stimulated with or without bFGF for 6.5 additional hours. (B) Densitometric quantification of cyclin D1 and cyclin E bands relative to the optical density of the corresponding β-actin bands. Results are the mean±SEM of 3 independent experiments; *P<.05 compared with none (basal levels of cyclins in the absence of basic fibroblast growth factor [bFGF] and protein kinase C [PKC]-modulating drugs) in a Student’s *t* test; #*P*<.05 compared with the other groups by 1-way ANOVA. (C) Graph shows the effect of 5 μM prostratin on both the basal proliferation of NPC in the absence of any added growth factors and on bFGF-induced NPC proliferation after 72 hours of culture. Prostratin alone significantly increased neurosphere area in the absence of added growth factors; also, a synergistic effect of prostratin and bFGF on neurosphere area enlargement was observed. Results are the mean±SEM of 3 independent experiments; ##*P*<.01 when compared with control by Student’s *t* test. (D) Representative phase-contrast photomicrographs of the neurosphere culture conditions explained in C. Calibration bar indicates 200 µm.

Next, the effect of prostratin treatment on cyclin expression was analyzed by Western blot. Cyclin E and cyclin D1 were upregulated in cultures stimulated by either bFGF or prostratin alone, and the combination of both treatments had a synergic effect on cyclin upregulation (Figure 3A-B). These results indicated a strong interaction between prostratin and bFGF signaling cascades on cyclin expression induction. But the fact that prostratin alone was able to induce the expression of cyclin D1 and E through PKC activation in growth factor-devoid cultures prompted us to consider whether this drug could, by itself, increase NPC proliferation. As shown in Figure 3C-D, prostratin alone, in the absence of any added growth factor, was able to induce a small but statistically significant increase on neurosphere area despite the fact that NPC are strictly dependent on growth factors for survival and proliferation in culture. A synergic effect of prostratin and bFGF on neurosphere growth was also evident in agreement with previous observations on cyclin expression patterns induced by the combination of both molecules.

### 12-Deoxyphorbols Isolated from *Euphorbia resinifera* Exert a More Potent Proliferative Effect Than Prostratin

**Figure 4.**
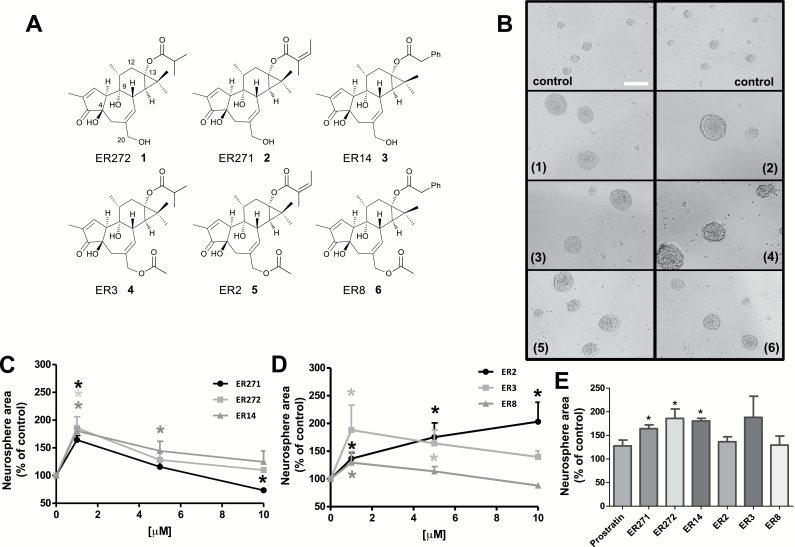
Effect of 12-deoxyphorbols on neural progenitor cell (NPC) proliferation. (A) Chemical structures of the 12-deoxyphorbols tested on proliferation assays are shown. Note that relevant carbon positions have been numbered on the structure of the first compound (ER272 [1]). (B) Phase contrast microscopy images of neurosphere cultures stimulated for 72 hours with basic fibroblast growth factor (bFGF) in the absence and presence of 1 µM 12-deoxyphorbols: ER272 (1), ER271 (2), ER14 (3), ER3 (4), ER2 (5), and ER8 (6). Calibration bar indicates 200 µm. (C) Effect of different concentrations of ER272 (1), ER271 (2), and ER14 (3) on neurosphere area. *P<.05 compared with control in a Student's *t* test. (D) Effect of different concentrations of ER2, ER3, and ER8 on neurosphere area. *P<.05 compared with control in a Student’s *t* test. (E) Comparative effect of prostratin, ER272 (1), ER271 (2), ER14 (3), ER3 (4), ER2 (5), and ER8 (6) tested at 1 µM on neurosphere area. *P<.05 compared with prostratin in Student’s *t* test.

Six 12-deoxyphorbols were isolated from *Euphorbia resinifera* plants, whose chemical structures have been resolved in the literature (Figure 4A): ER272 (1) (Schmidt and Evans, 1977); ER271 (2) (Schmidt and Evans, 1977; Fatope et al., 1996); ER14 (3) (Evans and Schmidt, 1976); ER3 (4) (Popplewell et al., 2010); ER2 (5) (Fatope et al., 1996; Fattorusso et al., 2002; Popplewell et al., 2010); and ER8 (6) (Evans and Schmidt, 1976; Fatope et al., 1996). The effect of these compounds on NPC proliferation was investigated using the neurosphere assay at 1, 5, and 10 µM concentrations. We found that all tested compounds increased neurosphere size (Figure 4B-D). Moreover, compounds 1 to 4 were able to increase neurosphere size 2-fold when used at 1 µM, suggesting these were the most potent PKC activators; however, higher concentrations decreased neurosphere size, probably because toxic dosage had been reached, similarly to what was observed with 10 µM prostratin. Compound 6 was the least potent compound, showing very little effect on neurosphere size (Figure 4 D). Regarding compound 5, it was the only one to induce a continuous concentration-dependent increment in neurosphere size, within the range of 1 to 10 µM (Figure 4 D), suggesting that this molecule could be the least toxic, the one with the wider therapeutic spectrum, and still reach maximum potency when applied at a high-enough concentration.

**Figure 5.**
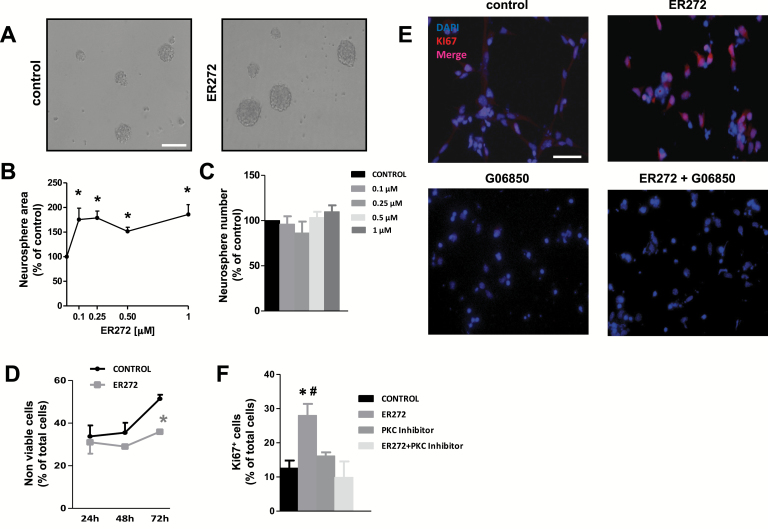
Effect of ER272 (1) on neural progenitor cell (NPC) proliferation. (A) Phase contrast microscopy images of neurosphere cultures stimulated with basic fibroblast growth factor (bFGF) in the absence and presence of 1 µM ER272 (1). Calibration bar indicates 200 µm. (B) Effect of different concentrations of ER272 (1) on neurosphere area. (C) Effect of different concentrations of ER272 (1) on neurosphere number. (D) Effect of 1 µM ER272 (1) on cell viability assayed by trypan blue exclusion. (E) Fluorescence microscopy images of NPC cultures stimulated with bFGF in the presence and absence of 1 µM ER272 (1) and/or 0.5 µM of the protein kinase C (PKC) inhibitor G06850. Cells were immunostained for Ki67 detection and nuclei were counterstained with 4',6-diamidino-2-phenylindole. (F) Quantification of Ki67^+^ cells in cultures described in E. *P<.05 compared with control in a Student’s *t* test; #*P*<.05 compared with the other groups by 1-way ANOVA.

**Figure 6.**
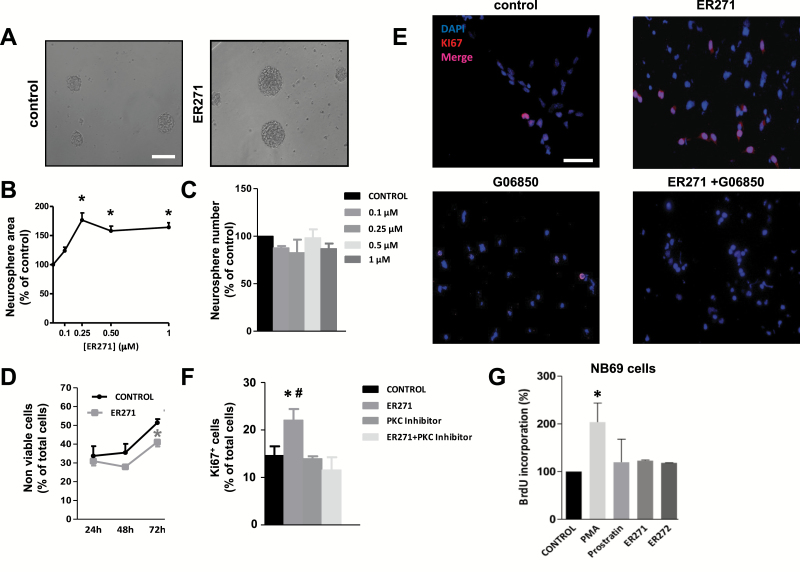
Effect of ER271 (2) on neural progenitor cell (NPC) proliferation. (A) Phase contrast microscopy images of neurosphere cultures stimulated with basic fibroblast growth factor (bFGF) in the absence and presence of 1 µM ER271 (2). Calibration bar indicates 200 µm. (B) Effect of different concentrations of ER271 (2) on neurosphere area. (C) Effect of different concentrations of ER271 (2) on neurosphere number. (D) Effect of 1 µM ER271 (2) on cell viability. (E) Fluorescence microscopy images of NPC cultures stimulated with bFGF in the presence and absence of 1 µM ER271 (2) and/or 0.5 µM of the protein kinase C (PKC) inhibitor G06850. Cells were immunostained for Ki67 detection and nuclei were counterstained with 4',6-diamidino-2-phenylindole. (F) Quantification of Ki67^+^ cells in cultures described in E. (G) Bromodeoxyuridine (BrdU) incorporation into NB69 human neuroblastoma cells (see supplementary file for procedure details) cultured for 48 hours in the presence of 8nM phorbol-12-myristate-13-acetate (PMA), 5 µM prostratin, 1 µM ER271 (2), 1 µM ER272 (1), or none (control). #*P*<.05 compared with the other groups by 1-way ANOVA.

From these results, a general conclusion inferred is that 12-deoxyphorbols 1 to 6 promoted NPC proliferation. Four compounds (1–4) exerted a more potent effect than prostratin on NPC proliferation at the smallest concentration used (1 µM), being ER272 the compound exerting the most potent effect (Figure 4E). Compounds ER272 and ER271 were next applied at lower, nanomolar concentrations. Using the neurosphere assay, we observed that concentrations of ER272 spanning from 0.1 to 1 µM increased neurosphere size to a similar extent, with no effect on neurosphere number (Figure 5A-C). Additionally, cell viability was improved to a significant extent after 72 hours in the presence of 1 µM ER272 (Figure 5D). Immunocytochemical experiments on adhered cultures revealed that ER272 induced a PKC-dependent 2-fold increase on the percentage of Ki67^+^ cells when used at 1 µM (Figure 5E-F). In summary, ER272 strongly induced NPC proliferation through PKC activation when used at concentrations 5 to 10 times lower than prostratin. The same experimental design was used to test ER271, and similar results were obtained, except that a slightly higher concentration of the drug (0.25 µM) was needed to exert maximal effects (Figure 6A-F). These results suggested that ER272 was more effective at activating PKC than ER271, notwithstanding that both were more potent than prostratin in promoting NPC proliferation.

### Prostratin, ER272, and ER271 Did Not Exert a Proliferative Effect on a Tumoral Neuroblastoma Cell Line

Tumor-promoting phorbols like PMA do not generate tumors by themselves but promote tumor growth following exposure to a subcarcinogenic dose of a carcinogen (they can thus be designated as co-carcinogens) (Goel et al., 2007). In an attempt to demonstrate that not only prostratin but also other 12-deoxyphorbols are nontumorigenic, we tested whether they were able to induce proliferation in cancerous cells. The effect of 8nM PMA, 5 µM prostratin, and 1 µM ER272 and ER271 on the proliferation of a neuroblastoma cell line (NB69) was assayed by quantifying BrdU incorporation. Unlike PMA, which increased 2-fold the proliferation of NB69 cells, neither prostratin nor ER272 or ER271 induced proliferation when added to NB69 cultures (Figure 6G). These results indicated that 12-deoxyphorbols, unlike PMA, exerted their proliferative effect specifically on NPC, without affecting neuroblastoma cells.

### In Vivo Administration of Prostratin, ER272, or ER271 Increases NPC Proliferation in Neurogenic Regions without Affecting Neuronal Differentiation

To study the relevance of PKC activation on NPC proliferation in vivo, we injected prostratin, ER272, and ER271 into adult mouse ventricles and studied their effect on neurogenesis. Single ICV injections of prostratin (4ng), ER272 or ER271 (0.8ng each), or vehicle were administered ipsilaterally. Proliferating cells were concurrently labeled by BrdU intraperitoneal injections for 3 days, when brains were fixed and subjected to immunohistochemical detection of BrdU in neurogenic areas. Proliferation was quantified as the number of BrdU^+^ cells found in the SVZ and DG, comparing the ipsilateral (injected) side of the brain with the contralateral side.

**Figure 7.**
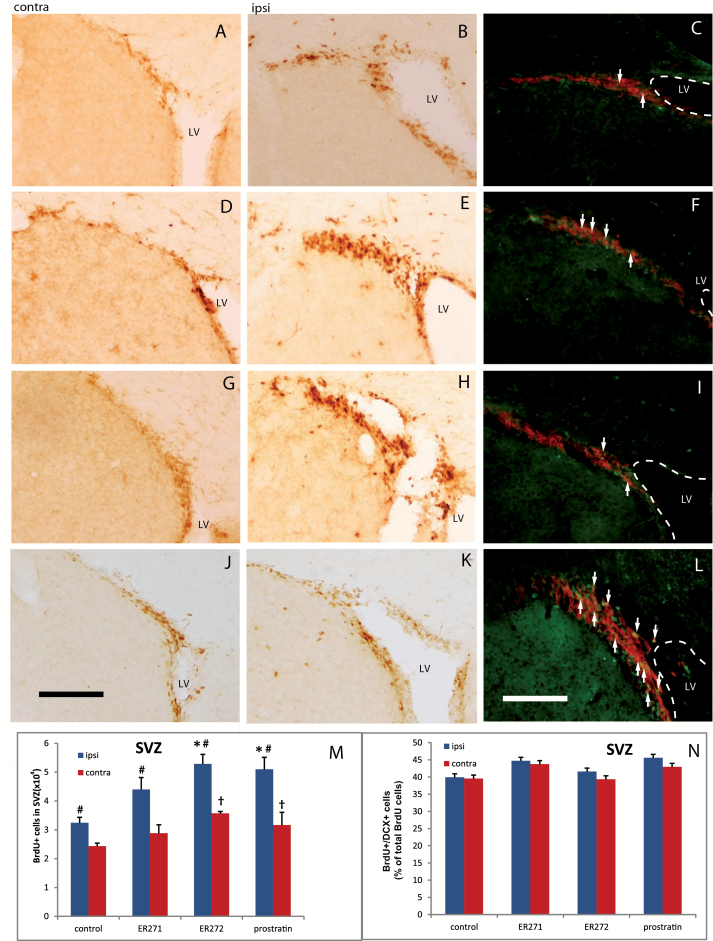
Effect of intracerebroventricular (ICV) administration of 12-deoxyphorbols on subventricular zone (SVZ) neurogenesis. Mice received single unilateral ICV injections of prostratin, ER271, ER272, or vehicle, and peritoneal injections of bromodeoxyuridine (BrdU) to label proliferating cells, as described in Methods. Photomicrographs show adult mouse brain coronal sections containing the SVZ. Left (A, D, G, J) and middle (B, E, H, K) column photomicrographs show contralateral and ipsilateral brain sections, respectively, processed for immunohistochemical detection of BrdU to label proliferating neural progenitor cells (NPCs). Right column photomicrographs (C, F, I, L) show ipsilateral sections processed for double immunofluorescence detection of BrdU and doublecortin (DCX), the latter being a cell marker for immature newly formed neurons; white arrows point to BrdU/DCX double-labeled cells, which represent a subpopulation of proliferating BrdU^+^ cells that are already committed to neuronal differentiation. (A-C) Vehicle-injected control mice. (D-F) Mice injected with ER272. (G-I) Mice injected with ER271. (J-L) Mice injected with prostratin. (M) Quantification of BrdU^+^ nuclei within the SVZ of mice that had received ICV injections of the indicated drugs; left bars correspond to the injected side of the brain (ipsilateral) and right bars correspond to the non-injected side of the brain (contralateral). (N) Quantification of the percentage of BrdU^+^ cells that express the early neuronal marker DCX; left bars (ipsilateral) and right bars (contralateral). contra, contralateral hemisphere; ipsi, ipsilateral hemisphere; LV, lateral ventricle. Statistics: #*P*<.05 comparing ipsilateral vs contralateral sides in a Student’s *t* test; *P<.05 when comparing different treatments to control ipsilateral in 1-way ANOVA test; †*P*<.05 when comparing different treatments with control contralateral in 1-way ANOVA test. Scale bar = 100 μm.

In the SVZ of vehicle-injected mice, a significant increase of BrdU^+^ cells was observed in the ipsilateral side compared with the contralateral side, indicating that the injection itself induced NPC proliferation (Figure 7A-B, M), probably due to the small lesion caused by the procedure. Treatment with prostratin significantly increased the total number of BrdU^+^ cells in both the ipsilateral and contralateral SVZs compared with their equivalent in vehicle-injected mice, demonstrating that in vivo administration of prostratin increased NPC proliferation in the SVZ of adult mice independently of, and further beyond, the increase due to procedural tissue damage (Figure 7J-K, M). Identically, treatment with ER272 significantly increased the number of BrdU^+^ cells in both the ipsilateral and contralateral SVZs (Figure 7D-E, M). However, ER271 injection did not induce a statistically significant increase on BrdU^+^ cells (Figure 7G-H, M).

To address whether in vivo treatment with 12-deoxyphorbols would alter the capacity of NPC to differentiate towards neuronal lineage, double immunostaining was performed in the SVZ of prostratin-, ER272-, and ER271-injected mice to detect the early neuronal differentiation marker doublecortin (DCX) within BrdU^+^ cells. As shown in Figure 7N, the percentage of BrdU^+^ cells that coexpressed DCX was not changed by any of these treatments (Figure 7C, F, I, L, N).

**Figure 8.**
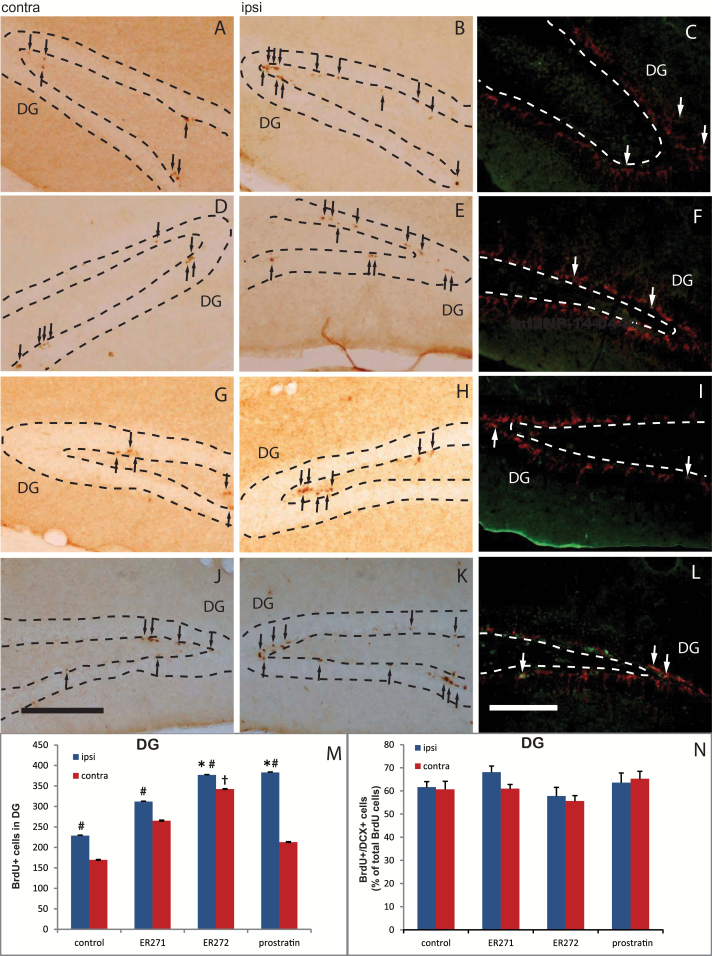
Effect of intracerebroventricular (ICV) administration of 12-deoxyphorbols on hippocampal neurogenesis. Mice received single unilateral ICV injections of prostratin, ER271, ER272 or vehicle, and peritoneal injections of bromodeoxyuridine (BrdU) to label proliferating cells, as described in Methods. Photomicrographs show adult mouse brain coronal sections containing the dentate gyrus of the hippocampus (DG). Left (A, D, G, J) and middle (B, E, H, K) column photomicrographs show contralateral and ipsilateral brain sections, respectively, processed for immunohistochemical detection of BrdU to label proliferating neural progenitor cells (NPCs); black arrows point to newly divided cells in the DG. Right column photomicrographs (C, F, I, L) show ipsilateral sections processed for double immunofluorescence detection of BrdU and doublecortin (DCX), the latter being a cell marker for immature newly formed neurons; white arrows point to BrdU/DCX double-labeled cells, which represent a subpopulation of proliferating BrdU^+^ cells that are already committed to neuronal differentiation. (A-C) Vehicle-injected control mice. (D-F) Mice injected with ER272. (G-I) Mice injected with ER271. (J-L) Mice injected with prostratin. (M) Quantification of BrdU^+^ nuclei within the DG of mice that had received ICV injections of the indicated drugs; left bars correspond to the injected side of the brain (ipsilateral) and right bars correspond to the noninjected side of the brain (contralateral). (N) Quantification of the percentage of BrdU^+^ cells that express the early neuronal marker DCX in the ipsilateral (left bars) or contralateral (right bars) side of the injection. contra, contralateral hemisphere; ipsi, ipsilateral hemisphere. #*P*<.05 when comparing ipsilateral vs contralateral sides in a Student’s *t* test; *P<.05 when comparing different treatments with control ipsilateral in 1-way ANOVA test; †*P*<.05 when comparing different treatments with control contralateral in 1-way ANOVA test. Scale bar = 200 μm.

In the DG, the injection procedure itself also had an effect on BrdU^+^ cell number (Figure 8A-B, M). Treatment with prostratin further increased NPC proliferation in the ipsilateral DG, but not in the contralateral DG (Figure 8J-K, M); the fact that prostratin’s effect did not reach the contralateral DG could be due to the wide distance between the site of injection (right lateral ventricle) and the DG on the other hemisphere. In contrast, ER272 increased the number of BrdU^+^ cells both in the ipsilateral and the contralateral DG (Figure 8D-E, M), indicating that this compound was not only the most potent in vitro, but also in vivo. ER271 did not have any effect on DG NPC proliferation (Figure 8G-H, M). Finally, none of these treatments affected the neurogenic capacity of NPCs within the DG (Figure 8C, F, I, L, N).

## Discussion

Neuronal replacement within the adult brain requires the generation of new neurons from NSCs. This process involves several steps: (1) activation and division of NSCs, giving rise to undifferentiated NPCs; (2) dynamic proliferation of NPCs; and (3) differentiation of NPC towards the neuronal lineage, which firstly requires production of committed neuroblasts that maturate into postmitotic neurons. Here, we have explored the capacity of new drugs with 12-deoxyphorbol structure to reinforce step number 2, that is, to expand NPC populations.

PKC activation promotes proliferation in different cell lines by cyclin upregulation (Watanabe et al., 1992; Dempsey et al., 2000; Black and Black, 2012), so we hypothesized that PKC activators would also promote NPC proliferation. Our results demonstrate that addition of PMA, a prototypical PKC activator, to bFGF-stimulated NPC cultures increased proliferation of NPC in a PKC-dependent manner.

Phorbol esters activate PKC by acting as diacylglicerol analogs, which help translocate PKC to the cell membrane, where enzyme function takes place. However, some phorbol esters like PMA are hardly metabolized by the cells, making them strong, irreversible drugs that hyperactivate PKC and amplify the efficacy of carcinogens (Goel et al., 2007). In contrast, other natural phorbols like prostratin are nontumorigenic PKC activators (Szallasi and Blumberg, 1991a, 1991b; Szallasi et al., 1992). Prostratin is a 12-deoxyphorbol currently investigated in preclinical trials for its potential use as a pharmacological agent. In our hands, prostratin was able to increase NPC proliferation in vitro to a similar extent as PMA without affecting the capacity of NPCs to differentiate towards the neuronal lineage right after treatment (supplementary Figure S1). Furthermore, prostratin was able to increase NPC proliferation even in the absence of any added growth factor (Figure 3C-D), but when added together with bFGF, a synergic effect on NPC proliferation was obtained. Prostratin not only increased NPC proliferation but also had a positive effect on cell survival, and both effects of prostratin disappeared when cells were co-incubated with the PKC inhibitor G06850 (Figure 2). In contrast to PKC activation, addition of G06850 alone had no decreasing effect on NPC proliferation (Figure 2C-D), suggesting that PKCs are not essential for basal bFGF-induced NPC proliferation, but, as demonstrated in here, these kinases can be pharmacologically activated to further increase NPC division.

We and others have previously shown that postnatal NPC proliferation is highly dependent on the activation of Erk1/2 and Akt signaling pathways (Sung et al., 2007; Torroglosa et al., 2007). Growth factors like EGF or bFGF can activate both pathways in these NPC, inducing a phospho-Erk1/2-driven upregulation of cyclins, like cyclin E, and a phospho-Akt-dependent phosphorylation and degradation of cyclin-dependent kinase inhibitors like p27 (Torroglosa et al., 2007; Rabaneda et al., 2008). Thus, the combination of cyclin upregulation and cyclin-dependent kinase inhibitor degradation results in cellular division. It has been reported that PKC isoforms, some of which are also activated by growth factors, can as well modulate the activity of Erk1/2 and Akt, although the type of modulation seems to differ depending on the cellular context and PKC subtypes involved (Black and Black, 2012; Kinehara et al., 2013). We have shown that Erk1/2 and Akt pathways were involved in prostratin-induced NPC proliferation and that both Erk1/2 and Akt are downstream effectors of prostratin-activated PKCs. This was inferred, since blockade of either pathway with specific inhibitors (UO126 and LY294002, respectively) ablated the effect of prostratin (supplementary Figure S2). Prostratin treatment also resulted in a PKC-dependent upregulation of cyclins D1 and E, both in the absence of growth factors and in synergy with bFGF (Figure 3A-B), suggesting an interaction between specific, still unidentified, PKC isoforms and proteins involved in the FGFR signaling cascade. An effect of prostratin on proliferation and cyclin expression in NPC had not been described before.

The effect of prostratin on adult brain neurogenesis was also analyzed in vivo. Our results showed that newly formed BrdU^+^ cells increased in both the DG and SVZ of adult mice after prostratin ICV administration. Endogenous neurogenesis is activated in response to brain damage (Parent et al., 1997; Liu et al., 1998; Jin et al., 2002; Nakatomi et al., 2002; Teramoto et al., 2003), generating NPCs and neuroblasts that eventually migrate to the damaged regions. In here, the small injury generated by the injections was enough to activate NPC proliferation in both the DG and SVZ, and treatment with 4ng of prostratin increased even further the proliferative response to the small injury in both neurogenic regions ipsilaterally. The effect of prostratin also reached the contralateral SVZ, but not the contralateral DG. These results suggest that prostratin might constitute a new drug to increase endogenous neurogenesis and facilitate neuronal renewal after brain lesions. In fact, neuronal commitment of NPC was not affected in vivo following prostratin administration (Figures 7N and 8N). The capacity of prostratin or related compounds to cross the blood brain barrier has not been tested, but given the lipophilicity of 12-deoxyphorbols, they should easily cross lipid membranes; this issue will be object of future investigations of our group.

Excessive NSC/NPC proliferation may result in NSC pool exhaustion. For instance, in Alzheimer’s disease mouse models, neurogenesis is highly increased at young ages (probably as a compensatory mechanism to neuronal damage) which, at last, results in neurogenic niche exhaustion at adult ages due to NSC depletion (Lopez-Toledano and Shelanski, 2007). Pharmacological expansion of NSC/NPC populations could similarly compromise their self-renewal capacity (Ramirez-Castillejo et al., 2006). However, we show in here that prostratin and PMA clearly have different effects on NPC self-renewal capacity. By analyzing the number of primary and secondary neurospheres formed we show that prostratin, but not PMA, may, on the long run, enhance self-renewal capacity increasing the pool of undifferentiated progenitors. Noteworthy, not all PKC activating drugs have the same outcome.

Nontumorigenic PKC activators, such as prostratin, have been enrolled as potentially safe drugs (Hecker, 1985). Therefore, we screened a library of natural compounds structurally similar to prostratin in the hope that new products with a more potent effect on neurogenesis than prostratin could be found. We were able to detect 2 compounds, ER271 and ER272, which were more potent than prostratin at inducing NPC proliferation. The lipophilic domain of phorbol esters, largely comprised of the C12/C13 diacyl moiety, is thought to be responsible for anchoring the phorbol-PKC complex to the plasma membrane, an absolute requirement for PKC activation. The ability of phorbol analogues to membrane-recruit PKC can be gradually decreased or completely abolished depending on the polarity and dimension of substitutions at these positions (Bertolini et al., 2003). There is a general agreement that only PKC agonists inducing irreversible PKC translocation to the cell membrane are endowed with tumor-promoting activity (Sanchez-Duffhues et al., 2011). Like prostratin, our selected 12-deoxyphorbols (1–6) from *E. resinifera* have short lipophilic C13 acyl chains and have no substitution at C12 (Figure 4A). Therefore, these compounds should not be tumor promoters. In relation to this, 12-deoxyphorbols (1–6) were assayed for their capacity to induce proliferation in neuroblastoma cells. In contrast to PMA, which was able to induce neuroblastoma cell proliferation, none of the 12-deoxyphorbols tested exerted a proliferative effect on these tumor cells, indicating that 12-deoxyphorbols, including prostratin and ER272, exerted their proliferative effect specifically on NPC, without affecting cancerous cells. Thus, the use of this type of compound in vivo may specifically facilitate NPC expansion after a CNS lesion without causing undesirable proliferation of other cell types.

ER272 and ER271, the most potent 12-deoxyphorbols in vitro, were also analyzed in vivo. Unilateral ICV injections of 0.8ng of ER271 did not significantly increase NPC proliferation in either the SVZ or DG, suggesting that this compound may be readily degraded or metabolized when injected in vivo. In contrast, unilateral injection of ER272 (0.8ng) significantly increased NPC proliferation in the SVZ and DG of both brain hemispheres (Figure 8D-E, M). The largest effect obtained with ER272 in vivo is in agreement with its highest potency in vitro but may also reflect other pharmacokinetic properties such as half-life in vivo or diffusion capacity, since ER272 reached as far as the distant contralateral DG. None of these compounds affected the capacity of NPCs to differentiate towards neuroblasts (Figures 7N and 8N).

The development of strategies to promote neurogenesis in damaged regions may constitute a successful approach to solve the clinical problems associated with neuronal loss after brain injury. We show here that activating PKC promotes proliferation of NPCs in vitro as well as in vivo. Furthermore, we are describing at least 5 new PKC activators, and further characterizing 2 of them, among which ER272 exerts the most potent effect. Identifying the specific PKC isoform targeted by these compounds is a crucial step to facilitate the development of drugs with a more specific and potent effect on adult brain neurogenesis.

## Statement of Interest

None.

## Supplementary Material

supplementary Methods
