# Supplementary material for: 12-Deoxyphorbols Promote Adult Neurogenesis by Inducing Neural Progenitor Cell Proliferation via PKC Activation
Source: Int J Neuropsychopharmacol. 2015 Jul 29;19(1):pyv085. doi: 10.1093/ijnp/pyv085 (PMC4772272; doi:10.1093/ijnp/pyv085)
Supplement: supplementary Methods [file Supplementary_File.pdf]

SUPPLEMENTARY METHODS AND FIGURES

SUPPLEMENTARY METHODS

Isolation of Natural Products

12-*D*eoxyphorbols, previously described in other species of the *Euphorbia* genus, were obtained by isolation and purification from methanolic extracts of the latex from *E. resinífera*. The methanolic fraction was subjected to silica gel column chromatography with an increasing gradient of ethyl acetate in hexane as mobile phase and monitored by TLC. Further semi-preparative and analytical HPLC yielded compounds with spectroscopic (^{1}H and ^{13}C NMR) and spectrometric (HRMS) data consistent with those described previously in the literature for 13-*O*-isobutiroyl-12-deoxiphorbol (ER272, **1**) (Schmidt and Evans, 1977), 13-*O*-angeloyl-12-deoxiphorbol (ER271, **2**) (Schmidt and Evans, 1977) (Fatope et al., 1996), 13-*O*-phenylacetyl-12-deoxiphorbol (ER14, **3**) (Evans and Schmidt, 1976), 20-*O*-acetyl-13-*O*-isobutiroyl-12-deoxiphorbol (ER3, **4**) (Popplewell et al., 2010) 20-*O*-acetyl-13-*O*-angeloyl-12-deoxiphorbol (ER2, **5**) (Fatope et al., 1996; Fattorusso et al., 2002; Popplewell et al., 2010), and 20-*O*-acetyl-13-*O*-phenilacetyl-12-deoxiphorbol (ER8, **6**) (Evans and Schmidt, 1976); (Fatope et al., 1996).

Stock solutions of these diterpenes were prepared in DMSO at a concentration of 5 mM, and were aliquoted and maintained at -20°C.

Purification by semi-preparative and analytical high-performance liquid chromatography (HPLC) was performed with a Hitachi/Merck L-6270 apparatus equipped with a differential refractometer detector (RI-7490). A LiChrospher® Si 60 (5 µm) LiChroCart® (250 mm_4 mm) column and a LiChrospher® Si 60 (10 µm) LiChroCart® (250 mm_10 mm) were used in isolation experiments with HPLC-grade solvents.

Silica gel (Merck-Millipore, Billerica MA USA) was used for column chromatography. Thin Layer chromatography (TLC) was performed on Merck Kiesegel 60 F254, 0.25 mm thick plates. ^{1}H and ^{13}C NMR measurements were recorded with Varian Unity 400 MHz, Agilent 500 MHz, and Varian Inova 600 MHz spectrometers with $SiMe_4$ as the internal reference. High-resolution mass spectra (HRMS) were recorded with a double-focusing magnetic sector mass spectrometer in positive ion mode, or with a QTOF mass spectrometer in positive ion ESI mode.

Proliferation assay for NB69 neuroblastoma cells

Proliferative responses of NB69 cells to different treatments were investigated using a colorimetric, BrdU incorporation-based, cell proliferation ELISA kit from Roche Diagnostics (Mannheim, Germany), by following manufacturer's instructions. BrdU (5-bromo-2'-deoxyuridine) is a thymidine analogue that cells incorporate into DNA during replication, and thus labels cells during the S phase of the cell cycle. NB69 cells were seeded on 96-well plates at a density of 5,000 cells per well and cultured for 48 h. BrdU was added 24 h after seeding. Subsequently, cells were fixed during 30 min at RT and incubated for 90 min at RT with an antibody against BrdU. Thereafter, plates were washed 3 times and incubated 30 min at RT with the substrate solution supplied with the kit. The colorimetric change was measured at 370 nm in an Elisa reader (PowerWave XS2, BioTek).

Immunocytochemistry to detect differentiation markers in NPC cultures after pre-treatment with PMA or prostratin

NPC were grown for 72 h in the presence of bFGF with or without the addition of prostratin (1 or 5 μM) or PMA (16 nM). At the end of treatments, the neurospheres formed were disaggregated and centrifuged to remove bFGF and drugs, and the cellular pellet was resuspended in defined medium plus 1% FCS (necessary to improve cell adhesion and survival) and seeded onto poly-L-ornithine-treated coverslips. Adhered cells were allowed to spontaneously differentiate in culture

for 72 h more, and then were fixed in 4% PFA and processed for immunocytochemistry to detect the immature neuron marker beta-III-tubulin and the astrocyte marker glial fibrilary acidic protein, or GFAP; nuclei were counterstained with DAPI (Sigma Aldrich). Primary antibodies used were: mouse monoclonal anti-beta-III-tubulin (StemCells Technologies, diluted to 1:1000) and rabbit anti-GFAP (DAKO, diluted to 1:5000). Secondary antibodies used were: Alexa Fluor 594-conjugated anti-mouse IgG (1:3000) and Alexa Fluor 488-conjugated anti-rabbit IgG (1:1000), both from Alexa.

Geribaldi-Doldan, Flores-Giubi, et al. Supplementary Figure S1

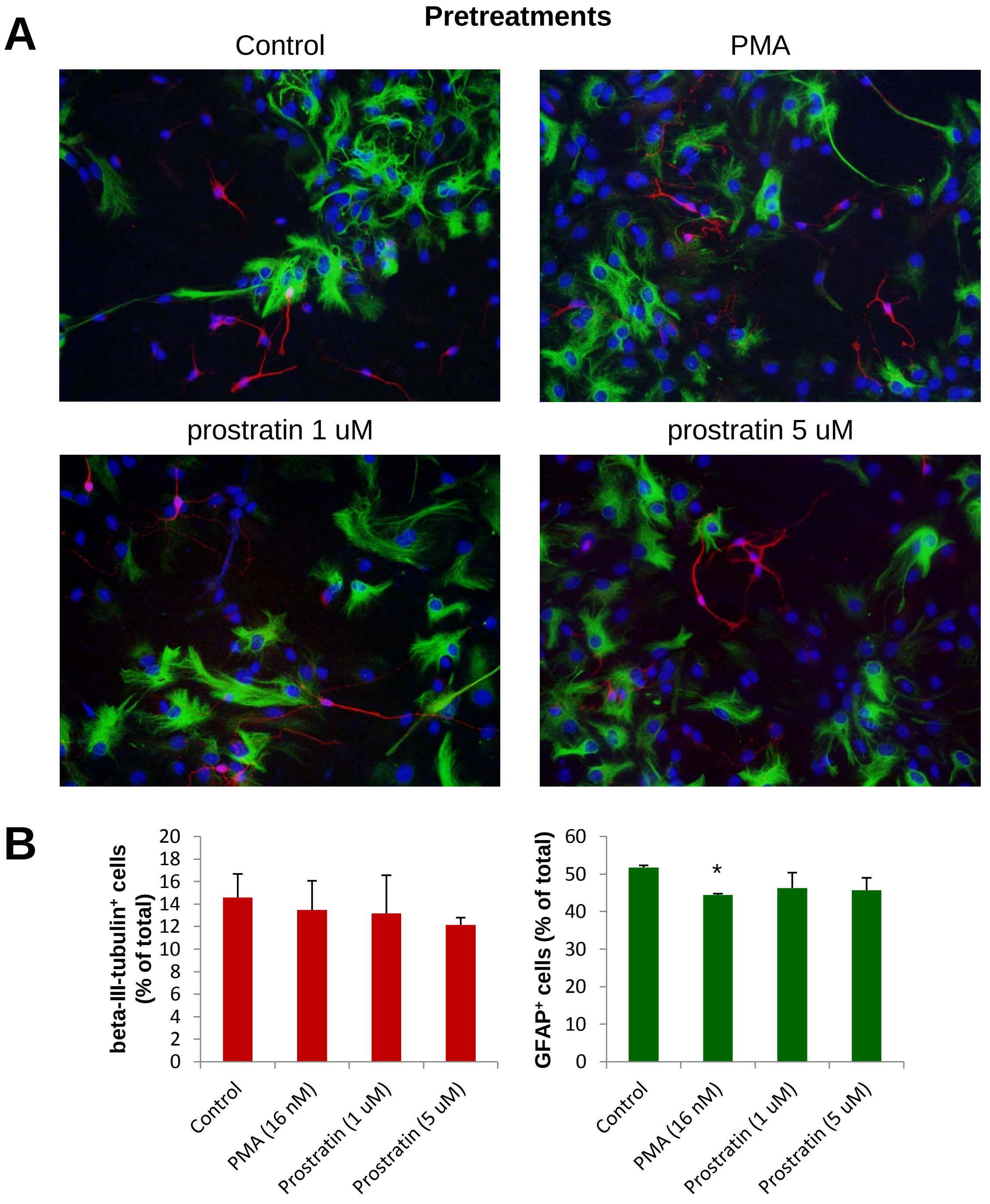

Figure S1. *In vitro* neuronal differentiation of NPC after treatment with PMA or prostratin. NPC were grown for 72 h in the presence of bFGF with or without the addition of prostratin or PMA, as indicated. The neurospheres formed were disaggregated and cells were adhered onto coverslips, in the absence of any added growth factor or treatment (see supplementary methods for details). After 72 h more in culture, cells were fixed and processed for immunocytochemistry to detect the neuronal marker beta-III-tubulin (red) and the astrocytic marker GFAP (green); nuclei were counterstained with DAPI (blue). Representative photomicrographs obtained after the different pretreatments are shown (**A**). Beta-III-tubulin^{+} and GFAP^{+} cells were counted and expressed as percentage of the total number of nuclei (**B**). The results show that pretreatment with the PKC-activating, proliferation-inducing drugs prostratin and PMA did not affect the capacity of NPC to commit neuronal lineage. A small effect of PMA on astrocyte differentiation was observed; (*) means $p < 0.05$ compared to control (Student's t test).

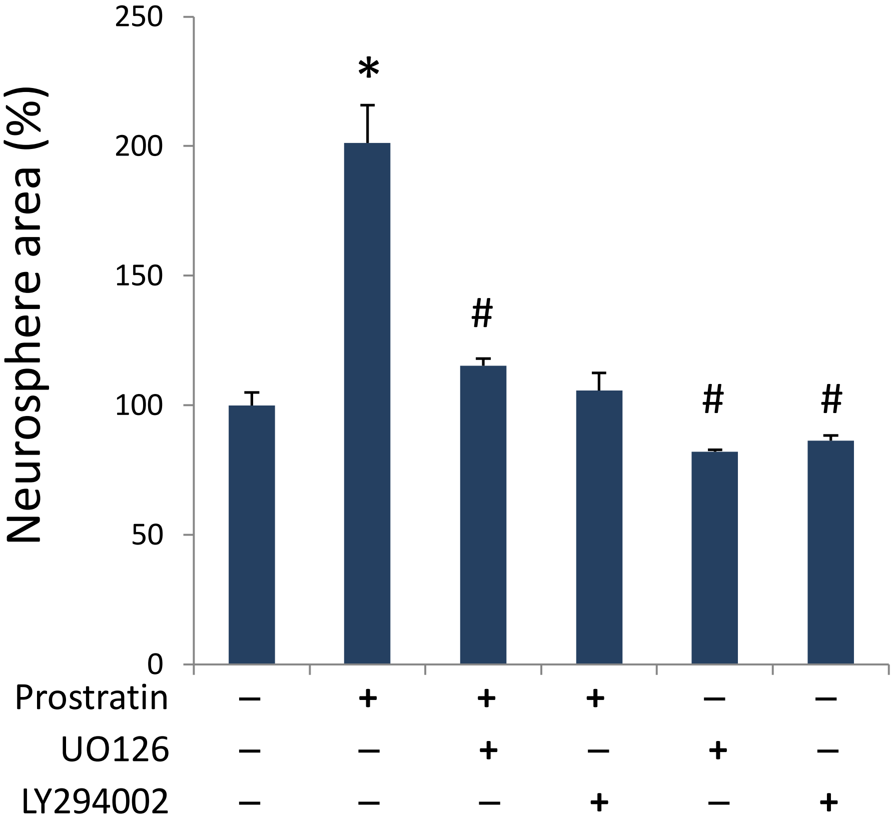

Figure S2. Erk1/2 and Akt pathways are both required for prostratin-induced NPC proliferation *in vitro*. A neurosphere assay was performed in bFGF-treated neural precursor cell cultures grown for 72 h in the absence or presence of 5 µM prostratin, with or without addition of the Erk1/2 pathway inhibitor UO126 (10 µM) or the Akt pathway inhibitor LY294002 (20 µM), as indicated on the graph. At the end of treatments, neurosphere area was measured and expressed as percentage of that in control cultures. (*) means $p < 0.01$ compared to rest of groups (Student´s t test); (#) means $p < 0.005$ compared to control (bFGF alone, first bar) (Student´s t test).
